# *Bothrops lanceolatus* Bites: Guidelines for Severity Assessment and Emergent Management

**DOI:** 10.3390/toxins2010163

**Published:** 2010-01-22

**Authors:** Dabor Resiere, Bruno Mégarbane, Ruddy Valentino, Hossein Mehdaoui, Laurent Thomas

**Affiliations:** 1 Centre Hospitalier Universitaire, Fort-de-France, Martinique, French West Indies, France; 2 Service des Urgences et de Réanimation polyvalente, Fort-de-France, Martinique, French West Indies, France; 3 Réanimation Médicale et Toxicologique, Hôpital Lariboisière, Paris, France

**Keywords:** *Bothrops lanceolatus*, Martinique, snake bite, severity, coaguloapthy, antivenom serum

## Abstract

Approximately 20-30 declared snakebite cases occurin Martinique each year. *Bothrops lanceolatus*, a member of the Crotalidae family, is considered to be the only involved snake. *B. lanceolatus*, commonly named “Fer-de-Lance”, is endemic and only found on this Caribbean island. Envenomation local features include the presence of fang marks, swelling, pain, bleeding from punctures, and ecchymosis. Severe envenomation is associated with multiple systemic thromboses appearing within 48 h of the bite and resulting in cerebral, myocardial or pulmonary infarctions. Diagnosis requires first of all identification of the snake. Coagulation tests are helpful to identify thrombocytopenia or disseminated intravascular coagulation. A clinical score based on 4 grades is helpful to assess envonimation severity. A specific monovalent equine anti-venom (Bothrofav^®^, Sanofi-Pasteur, France) to neutralize *B. lanceolatus* venom is available. Its early administration within 6h from the biting in case of progressive local injures, general signs or coagulation disturbances is effective to prevent severe thrombosis and coagulopathy. Its tolerance is considered to be good. Despite an increasing incidence of bites, no deaths have been recently attributed to *B. lanceolatus* in Martinique, probably due to the currently recommended strategy of early antivenom administration when required.

## 1. Introduction

**Figure 1:**
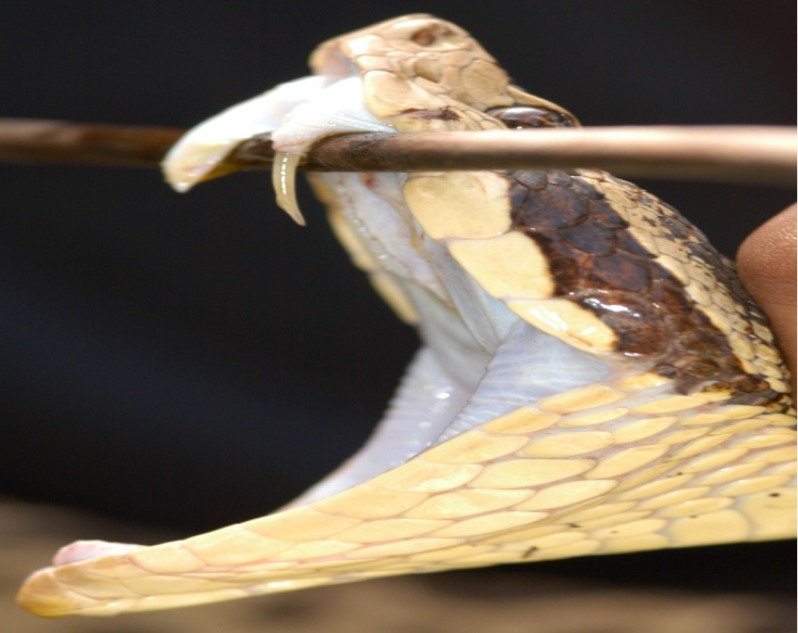
*Bothrops lanceolatus.*

*Bothrops lanceolatus* (Order: Squamata; Suborder: Serpentes; Family: Crotalinae; Figure 1) is endemic in Martinique French West Indies. It is commonly called Martinique lancehead (“Fer-de-Lance”) and Martinican pit viper [1,2]. Each year, approximately 20-30 declared cases of snakebites occur in this island [3,4]. *B. lanceolatus*, which is not found elsewhere in the world, is the only indigenous snake present in Martinique. Interestingly, the species was depicted on the unofficial flag of Martinique. The reason why it has flourished there and not on the other Caribbean islands has never been satisfactorily explained. No *B. lanceolatus* subspecies are currently recognized.

The Island of Martinique is localized in the eastern part of the Caribbean. To the northwest lies Dominica and to the south St Lucia. The Martinican population is approximately 402 000 inhabitants and live in the 1,128 km2 island. Martinique is an overseas department of France and a part of the European Union. In France, an estimated 3,000 venomous snakebites occur per year. Although *B. lanceolatus* is not found naturally outside of Martinique, it may be imported into zoos, museums, and private collections to other regions [3,5].

In the past 20 years, the incidence of *B. lanceolatus* bites has increased in Martinique while its mortality rate decreased, probably because of the introduction in 1993 of an effective specific equine monovalent antivenom. The purpose of this review is to provide guidelines to help physicians in the emergency room and intensive care unit assessing *B. lanceolatus* envenomation severity and to highlight the latest achievements regarding management.

## 2. Envonimation Pathophysiology

Bothrops species snakebites account for the majority of envenomations in South and Central America. Envenoming by Bothrops sp. is characterized by a highly complex pathophysiology, including local effects like oedema, pain, haemorrhage, and necrosis and systemic effects like coagulation disturbances, thrombosis, and renal failure. Bothrops venom generally contains several digestive enzymes and spreading factors, resulting in these local and systemic injuries. The major enzymes are hemorrhagic snake venom zinc-dependent metalloproteinases called “hemorrhagins”, that produce haemorrhage by hydrolyzing proteins in the basal lamina of the capillary vessels [6]. Another important component is the phospholipase A2, responsible of oedema-formation, myotoxicity, and additional anticoagulant effects.

*B. lanceolatus* venom induces comparable local and systemic effects with a predominant prothrombotic but a rare necrosis and hemorrhage profile. Proteomic studies revealed the following content in *B. lanceolatus* venom: acidic phospholipases A2, serine proteinases, l-amino acid oxidases, zinc-dependent metalloproteinases, and a specific C-type lectin-like molecule [7,8,9]. The oedematogenic and hemorrhagic activity of *B. lanceolatus* venom is thermolabile [10]. Compositional differences with other venoms among closely related species from different geographic regions are due to evolutionary environmental pressure acting on isolated populations. Although *B. lanceolatus* venom contains approximately the same amount of metalloproteinases than other Bothrops species, its metalloproteinase subgroups are different with particular biochemical and pharmacological characteristics, like a lesser in vitro gelatinolytic activity [11]. The acidic phospholipase A2 consists in two different isoenzymes characterized by a progressive increase in the rate of in vitro hydrolysis activity, which seems mainly dependent on the physical state of phospholipids [12]. In mice, *B. lanceolatus* venom produces dose- and time-dependent local oedema and myotoxicity but is devoid of coagulant and defibrinogenating effects. The characteristic thrombotic effect described in human envenoming is not reproduced in the mouse model. Oedema is partially dependent on a “hemorrhagin” and involve the release of arachidonic acid metabolites (by either cyclooxygenase or lipooxygenase), bradykinin, histamine, and serotonin [13]. Both polyvalent Crotalinae and monospecific *B. lanceolatus* antivenom were shown to immunodeplete *B. lanceolatus* venoms in vitro and to be fully effective in mice in neutralizing the lethal, hemorrhagic, oedema-forming, myotoxic, and indirect hemolytic activities of the venom [7,14,15]. 

*B. lanceolatus* is the only snake in the world whose venom produces significant systemic thrombotic complications [2,16]. Development of local thrombosis phenomena is thought to be a result of endothelium injury induced by direct action of the venom on the vessels. When added to human-citrated plasma, the venom of *B. lanceolatus* has no coagulant effect, even at concentrations as high as 100 µg/mL [14]. This is a unique feature that characterizes *B. lanceolatus* venom vis-à-vis other Bothrops venoms. Interestingly, the monospecific *B. lanceolatus* antivenin is devoid of neutralizing capacity against the procoagulant and defibrinating activities of heterologous Bothrops snake venoms in mice [14].

## 3. Clinical Features

Envenomation by *B. lanceolatus* leads to swelling and pain, and occasionally to systemic signs and/or coagulopathy. Severity of envenoming by *B. lanceolatus* depends on the injected amount of venom as well as on the victim’s conditions such as age, body weight, immunity, and past medical history, as described for other crotalidae bites [17]. The venom isinjected into the subcutaneous tissues via hollow movable fangs located in the snake anterior mouth. Occasionally intramuscular and rarely intravenous injection may occur. 

Clinically, local effects such as pain, bleeding from the fang punctures, swelling, erythema, ecchymoses, and bullae may occur. Local envenomation may increase over time, resulting in serious complications such as blistering, local necrosis, abscesses, and extensive swelling involving the whole bitten limb and the trunk. Severe envenomation is regularly associated with systemic multifocal thrombotic complications, usually occurring within two days after the bite. Rapidly progressive swelling is usually indicative of severe envenomation. Hypotension/hypertension, tachycardia, muscle fasciculation, weakness, lethargy, difficulty of breathing, chest pain, and (near)-syncope are predictive of further life-threatening complications. However, complications may develop in patients who initially have signs of only moderate envenomation associated with normal results of blood coagulation tests apart from decreased platelet counts [5,18]. 

Approximately 30 to 40% of envenomed cases by *B. lanceolatus* bites lead to multiple vascular obstruction, in which the exact mechanism remains unknown [2]. Vascular thrombosis at distance from the site of the bite may occur despite the hypocoagulable conditions due to disseminated intravascular coagulation. This is a unique situation in the snake world, closely related B. caribbaeus on the neighbouring island of St. Lucia. It differs dramatically from the systemic bothropic syndrome observed in Central and South America, characterized by the development of incoagulable blood and spontaneous systemic bleeding [2,3]. Thromboses involve cerebral, myocardial, and pulmonary arteries, occur despite heparin therapy, and lead to death or major functional sequelae in approximately 25% of the cases [5,18,19,20,21,22,23]. Of fifty adult snake bite cases hospitalized between June 1991 and August 1994 in Fort de France University Hospital, eleven developed serious thrombotic complications following envenomation, despite early preventive anticoagulant therapy including pulmonary embolism (two cases), cerebral infarction (six cases), myocardial infarction (one case), and cerebral and myocardial infarctions (two cases) [5]. Consistently, a 2.6%-prevalence of cerebrovascular complications was related to Bothrops spp. bites [21] Intracranial haemorrhages were more frequently assessed than cerebral infarcts, in contrast to *B. lanceolatus*, thus enhancing its particularities. Interestingly, a fatal case with diffuse thrombotic microangiopathy causing multiple cerebral, myocardial, and mesenteric infarctions at autopsy was reported [24]. Multi-focal thrombotic microangiopathy was diagnosed with intimal-medial dissection by thrombi extending from foci of endothelial damage in small cerebral, myocardial, pulmonary, mesenteric, and renal arteries and arterioles, suggesting the presence in *B. lanceolatus* venom of von Willebrand factor activators (like in inherited thrombotic thrombocytopenic purpura and hemolytic uremic syndrome) or vascular endothelial growth factor-type factors of the kind demonstrated in several Viperidae venoms.

## 4. Envenomation Diagnosis

**Figure 2:**
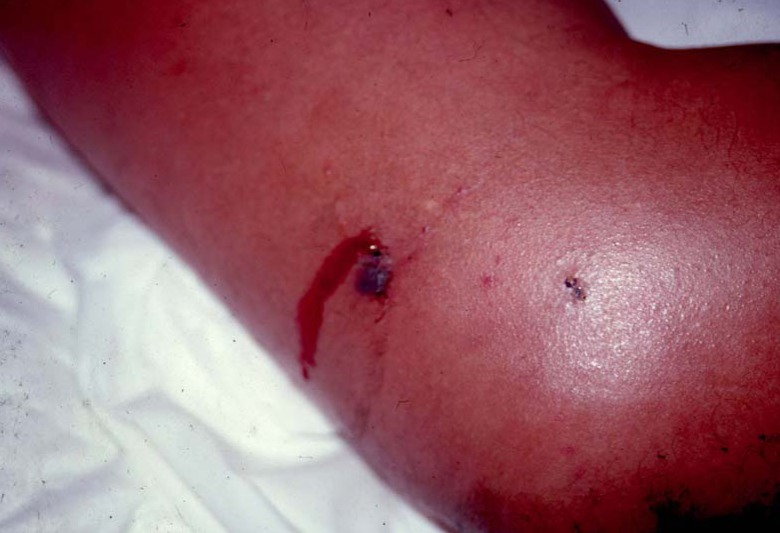
Clinical aspect of *Bothrops lanceolatus* bite with an extensive inflammatory swelling surrounding the fang punctures.

Diagnosis of *B. lanceolatus* envenomation is based on the circumstances of the bite and identification of the snake, when possible, as well as the presence of bleeding, swelling, and pain from the fang punctures (Figure 2). Envenomation severity is assessed using an updated clinical scoring (Table 1). On admission, routine laboratory tests are recommended, typically including blood cell count, complete coagulation tests (e.g., at least platelets, prothrombin time, activated partial thromboplastin time, serum fibrinogen, and clotting factors), and serum chemistry tests (e.g., sodium, potassium, chloride, bicarbonate, blood urea nitrogen, creatinine, calcium, and phosphorus). Plasma creatine phosphokinase is mandatory to rule out rhabdomyolysis. 

**Table 1:** Updated severity score after *Bothrops lanceolatus* bite (evidence of fang marks) and subsequent doses ofBothropfav^®^ antivenom serum to be infused.

**Grade**	**Severity ^a^**	**Symptoms**	**Dose of antivenom ^b^**
1	Minor	No swelling	None
		No pain	
		No general signs	
2	Moderate	Local swelling confined to 2 segments of the bitten limb	40 mL
		Moderate pain	
		No general signs	
3	Severe	Regional oedema: extension of swelling beyond 2 segments	60 mL
		Persistent and resistant pain to analgesics	
		No general signs	
4	Major	Swelling spreading to the trunk	80 mL
		General signs (vomiting, headache, abdominal or chest pain)	
		Hypotension	
		Isolated thrombocytopenia	
		Disseminated intravascular coagulation	

^a^ Severity is defined by at least one confirmed item.

^b^ To be given by intravenous infusion or by electrical syringe, diluted in isotonic saline with a flow at about 10 to 20 mL/h. Potential allergic reaction should be considered in all patients.

Coagulopathy commonly occurs with *B. lanceolatus* envenomation, although clinical bleeding is uncommon. Thrombocytopenia most often characterize this coagulopathy, while defibrination is more exceptional. Venom-induced thrombocytopenia may exist in association with or independently of defibrination. Defibrination is manifested by low serum fibrinogen, elevated prothrombin time, and elevated fibrin split products. More than half of the patients envenomed by *B. lanceolatus* have thrombocytopenia while rare patients develop disseminated intravascular coagulation [5,18,25]. Thrombocytopenia, minimally reduced prothrombin, normal activated partial thromboplastin time, and elevated fibrinogen concentration are typical features in victims of *B. lanceolatus* envenomation who will further develop thromboses [24]. As no evidence of coagulopathy apart from thrombocytopenia is generally observed, thrombosis is thought to result not from a direct procoagulant effect of the venom but from a vasculopathic origin (e.g., activation of the vascular endothelium) [1]. The close monitoring of platelets count is warranted over the first few days. All patients diagnosed with thrombocytopenia have been upgraded to a grade 4 envenoming (Table 1).

An enzyme-linked immunosorbent assay (ELISA) was developed to measure venom antigen levels in the serum of patients bitten by *B. lanceolatus* [25]. A good correlation was found between blood venom levels and clinical indicators of envenomation severitv. Serum venom levels significantly increased with the grade of severity. However, venom antigens could not be detected in the serum of 54% of patients who showed clinical signs of envenomation. No venom was detectable in blood samples taken after completion of serotherapy. In contrast, venom concentrations ≥ 15 ng/mL were observed in all patients with progressive aggravation of swelling despite the use of early antivenom therapy. 

Otherwise, plain radiographs may depict teeth or fangs retained in the wound. Chest X-rays, electrocardiography, and serum troponin I-c measurement may be indicated on the basis of envenoming severity. When suspecting thrombotic or embolic complications, additional radiological examinations may be useful. A head CT-scan and if available, a MRI should be considered in patients with headaches or altered level of consciousness if presenting severe thrombopenia or rarely any other coagulopathy, to rule out cerebral infarctions or haemorrhages.

## 5. Prehospital and Emergency Department Cares

After a bite from *B. lanceolatus*, the victim should be moved and placed at rest and transported to the emergency department of the nearest hospital [26]. The ABC’s (airway, breathing, and circulation) should be first addressed. General support of airway, breathing, and circulation should be given per advanced cardiac life support (ACLS) protocols with oxygen, two large bore intravenous lines, fluid challenge, and close monitoring. Minimizing patient’s activity is recommended when possible. Removing jewellery and tight-fitting clothes should be done in anticipation of swelling. After stabilization, a rapid detailed history should be obtained with a complete physical examination. The bite should be examined carefully to rule out fang, scratches, oedema, erythema, and ecchymoses. A pen to mark and time the border of advancing oedema is often enough to gauge progression. Subsequently, repeated measurements of the site of the bite should be documented until local progression of the swelling stops. Any tourniquet or constriction should be removed quickly if already placed as first aid. This is not recommended in Martinique, where immediately available anti-venom should be initiated in the emergency room if required.

Management in the emergency department includes initial non-specific supportive measures. Usually, victims require adequate intravenous hydration to correct hypotension and electrolyte disorders. Tracheal intubation and mechanical ventilation are mandatory in case of coma, severe collapse, cardiac arrhythmia, or respiratory distress. Shock is treated with fluids, followed by vasopressors to improve haemodynamic function or inotropic drugs to counteract conduction disturbances. However, such life-threatening presentations remain exceptional with *B. lanceolatus* envenomation. Analgesics and tetanus prophylaxis are systematic. In addition, grade 3 and 4 victims should receive empiric antibiotics, as more than one-third of these patients present with a primary bacterial infection [5,18,22]. A suggested association of cefotaxime, gentamycine, and metronidazole appears adapted to the bacteria found in the snake oral cavity, including A. hydrophila, M. morganii, P. vulgaris, and Clostridium sp. [5]. Prophylactic antibiotics may also be discussed in the other patients, according to the rapidity of local envenoming signs, due to the high risk of wound infection. In contrast, systematic administration of corticosteroids, histamine H1- and H2-receptor blockers, and heparin has not been evidenced [5]. All *B. lanceolatus* victims should be admitted, either to the emergency department for continuous and close observation for a minimum of 24h before discharge or to the intensive care unit if treated with antivenom.

## 6. Antivenom Therapy

The most important variable affecting the severity and outcome of envenoming from most viperid and crotalid snakes is the time elapsed between the bite and treatment initiation with a specific antivenom [2]. Usually, when considering any antivenom in snake-bites, the risk of adverse reactions must be weighed against the benefits of reducing venom toxicity. A purified monospecific antivenom serum for *B. lanceolatus* toxin neutralization is available since 1993 in Martinique [5,14].Bothrofav® is manufactured by Sanofi-Pasteur, Lyon, France. It has been obtained from horses hyperimmunized with *B. lanceolatus* venoms. It contains 97% antivenom F(ab)2 (bivalent antigen-binding fragment) and 3% antivenom Fab (antigen-binding fragment). Alternatives (e.g., different other types of antivenom) should be considered as well if available.

Initial strategy was to reserve antivenom for the most severe cases and use anticoagulants to prevent thromboses in all other patients. However, this approach was modified after the observation of serious thromboses in patients with moderate poisoning [5]. A prospective study assessed that administration within 6 h of the bite is the only effective treatment in preventing severe thromboses, when compared with death or major sequelae in 25% of untreated patients [22]. Moreover, it was shown to significantly reduce the length of hospital treatment. General recommendations for the management of crotalid envenomations proposed in 1983 by Reid & Theakston [26] are valid for *B. lanceolatus* [25]. Because clinical indicators of severity are often associated with high serum levels of venom, specific antivenom serotherapy is recommended to always be given to the cases of envenoming determined to be the most serious on clinical grounds. The presence of such indicators in the course of crotalid envenomation, even if labelled as “moderate” calls for immediate treatment with an appropriate minimum dose of specific antivenom, without waiting for signs of more severe envenomation. 

Antivenom administration is indicated in case of progressive signs of envenomation or imminent risk of acute complication. Ideally, it should be intravenously infused less than 30 min after hospital admission: its effectiveness is a function of how rapidly it is administrated [18,27]. It remains efficient even 4 to 5 days after the biting. Its dose regimen should be adapted to the envenomation severity, e.g., to the development of oedema and pain intensity (grade 2 to 4, Table 1). In bitten children, the regimen is unchanged whatever their weight is but may require continuous infusion using electrical syringe to limit the infused volume. This clinical attitude is based on the postulate established with pit viper bites, that signs are a reflection of the amount of venom injected [4]. The recommended doses were recently increased in comparison to those in the 90s, to be at least 40 to 80 mL, intravenously delivered at 10-20 mL per hour infusion rate (Table 1) [19]. Treatment duration is approximately 2-8 hours, till control of envenomation syndrome is achieved. As grading envenomation is a dynamic process, additional doses should be administered, based on the patient's clinical course (e.g., for recurrent progressive swelling or coagulopathy). 

In a recent study, among a total of thirty-three patients who did not receive antivenom serum or who received it eight hours after being bitten, fourteen (42%) developed severe thrombotic complications such as cerebral infarctions, myocardial infarctus, myocardial and cerebral infarctions and pulmonary embolisms [5,18]. Four of the fourteen patients who were not treated with the antivenom, died. Interestingly, in seventy patients who received the antivenom within 6 hours of being bitten, no thrombotic complications were observed. Thus, antivenom serum has to be given in the first six hours prior to the bite, as a key for success. However, despite this strategy, three patients bitten by *B. lanceolatus* were recently reported to have developed cerebral infarctions [19]. These 3 patients received the antivenom within the required time. Several causes were suggested to explain the treatment failure, including variability in the venom composition or loss of activity of the antibodies produced, in the 90s. 

Researchers are now working to identify the main causes for treatment failure. Sanofi-Pasteur announced a reduction of the antivenom power of their marketed serum up to 20-fold the lethal dose 50% per millilitre. To our opinion, all *B. lanceolatus* venom antigens may have not been represented in the initial venom pool which was derived from only 7 snakes used to immunise horses for antivenom production. The World Health Organisation currently recommends a pool from at least 50 snakes of each species to obtain a reliable antivenomen serum [28]. Thus, antivenom serum should be re-improved while probably including activity against Bothrops caribbaeus which shares a monophyletic group with *B. lanceolatus* and whose venom produces a similar thrombotic syndrome [16]. However, the manufacture of such an antivenom represents a great challenge for the future.

Early intravenous infusion of antivenom serum is well-tolerated [22]. Undesirable reactions may occur with this equine antivenom, including immediate anaphylactic reactions, anaphylactoid reactions (like vomiting, rash, pruritus, and bronchospasm), and serum sickness responding to steroids. However, their incidence is rare and their severity mild. If the side-effect is not life-threatening, antivenom serum infusion should be first interrupted and then reintroduced up to the initially advised dose. Thus, as Bothrofav® appears safe and well-tolerated, it should be indicated even if the envenomation is moderate. Moreover, it may be considered as a preventative measure even if any signs of envenomation exist. Waiting for the envenomation to get worse is not recommended as permanent injury could result.

## 7. Conclusions

*Bothrops lanceolatus*, notoriously named “Fer-de-Lance”, is the only endemic snake in Martinique. It is responsible of about 20-30 declared bites per year. Envenomation generally leads to swelling and pain, while occasionally, systemic signs and/or coagulopathy may appear. Severe multifocal vascular thromboses may result in life-threatening symptoms leading to permanent disabilities or death. An equine monovalent antivenom serum (Bothrofav®) was shown to be safe and effective for the treatment of *B. lanceolatus* bites when used within 6 hours. However, in recent cases, thrombotic stroke occurred despite antivenom early administration, questioning the necessity to obtain an improved antivenom.
